# A roadmap to equity in pandemic preparedness, prevention and response

**DOI:** 10.7189/jogh.14.03031

**Published:** 2024-08-23

**Authors:** Pierre Walckiers, Christine Frison, Sylvain Aubry

**Affiliations:** 1 Law Faculty, University of Louvain, Louvain-la-Neuve, Belgium; 2 Law Faculty, University of Liège, Liège, Belgium; 3 Government and Law Research Group, University of Antwerp, Antwerp, Belgium; 4 European Research Council – Starting Grant DecoLawBiodiv project; 5 Federal Office for Agriculture, Bern, Switzerland; 6 Department of Plant and Microbial Biology, University of Zürich, Zürich, Switzerland

Sometimes relegated to ‘low’ politics, global health lacked substantial and coherent international attention for a long time [1]. The emergence of the coronavirus disease 2019 (COVID-19) pandemic may have reversed this situation, underlining the urgency of a global health policy and fostering a shared commitment to bring equity within the global health system [2]. The most significant shift may be opening formal negotiations and creating a Pandemic Treaty, a new internationally legally binding instrument dedicated to pandemic prevention and response [3]. Indeed, the COVID-19 pandemic revealed a lack in the global governance of pathogen genetic resources – access to pathogen samples and data had been delayed, and the various exchanges that eventually occurred during the pandemic were mostly outside any international regulation [4]. A new legally binding instrument would address this gap – more efficient access and a fairer share of the benefits that may derive from using the pathogen genetic resources.

At a Special Session of the World Health Assembly (WHA) of December 2021, an intergovernmental negotiating body (INB) was established by the World Health Organization’s (WHO) member states to draft a convention, agreement, or any other international instruments on pandemic prevention and response, formally referred to as the WHO Convention, Agreement or Other International Instrument on Pandemic Prevention, Preparedness and Response (WHO CA+), and hereafter referred to as the Pandemic Treaty [5]. Based on a conceptual zero draft introduced in November 2022 (A/INB/3/3), various intermediary drafts have been published during the two-year process. We document and comment here on the evolution of the process through the various versions of the draft published and focus on three key time points – October 2023 (A/INB/7/3) [6], March 2024 (A/INB/9/3) [7,8] and final draft communicated to the 77th WHA between May 27 and June 1 2024 (A/INB/9/3Revision 1) [9]. The 77th WHA decided to extend the initial mandate of the INB for an additional year to finalise a Treaty (A77/10 and A77/A/CONF/15). Therefore, it is an ideal time to evaluate how equity and access, and benefit-sharing principles have primed the CA+ discussions. From the beginning, some elements were central to reaching an agreement, like the modalities for technology transfer, vaccines and other pandemic countermeasures, the definition of a new pathogen access and benefit-sharing (PABS) system, and a dedicated financing strategy [2]. Most of the discussions have crystallised around the various versions of Article 12 (PABS System) to determine the modalities, i.e. the degree of multilateralism, to be integrated into access and benefit-sharing in a pandemic.

While most parties advocate for norms of solidarity, fairness, transparency, inclusiveness, and equity as means to address the shortcomings of the COVID-19 international response [2,3], the efforts to design a new Pandemic Treaty are taking place in a time where the credibility of the global access and benefit-sharing (ABS) mechanism is being tested – a ‘make-or-break’ moment. The Convention on Biological Diversity (CBD) recognises the sovereignty of states over their genetic resources (CBD, Article 15) and imposes rules on ABS for their use. Therefore, ABS aims to ensure that the countries and communities providing these resources and knowledge are adequately recognised and rewarded for their contributions to biodiversity conservation [10,11]. The ABS mechanism was further developed through its Nagoya Protocol (NP) to shape the CBD implementation [12]. Benefit-sharing can include financial payments, technology transfer, capacity-building initiatives, and sharing of research results (NP, Article 5) [2]. The CBD and its NP are often considered umbrella treaties for the entire ABS regime complex [13].

Noteworthy, human pathogens, including pandemic ones, may fall under the scope of the CBD and the NP. Indeed, the CBD defines genetic material as ‘any material of plant, animal, microbial or other origin’ (CBD, Article 2), therefore explicitly including microbes. The NP also recognises ‘the importance of genetic resources to public health’ and ‘the importance of ensuring access to human pathogens for public health preparedness and response purposes’ in its preamble and Article 8. That renders the governance of pathogens, particularly of pathogen genetic resources (paGR), complex and fragmented. In most cases, paGR belong to the pool of genetic resources that falls under the national sovereignty of their country of origin and which access is regulated on a bilateral basis.

Meanwhile, the Pandemic Influenza Preparedness Framework (PIP Framework) may be one of the most advanced and complex ABS systems dealing with pathogens [14]. It was adopted in 2011 as an ad hoc and soft law mechanism of ABS and governance specifically dedicated to pandemic influenza strains [15]. In principle, sharing pandemic influenza biological material should be guaranteed through two sets of Standard Material Transfer Agreements (SMTAs) binding under private law terms for providers and users [15,16]. Under SMTA 1, member states accept the provision of human influenza viruses of pandemic potential to the Global Influenza Surveillance and Response System for monitoring (PIP Framework, Article 5.4.1 and Annex 1) [15]. Then, through SMTA 2, member states agree to transfer their materials to third parties and entities outside the WHO, such as recognised GISRS networks, academic laboratories, diagnostic and vaccine manufacturers, etc. (PIP Framework, Article 5.4.2 and Annex 2). As negotiated under SMTA 2, third parties should share with the WHO certain benefits in case of an influenza pandemic, e.g. access to vaccines and antivirals, granting royalty-free licenses to a vaccine in developing countries, etc. (PIP Framework, Annex 2, Article 4.1.1.) [15,17]. These two SMTAs have been hailed as innovative means of generating binding contracts favouring ABS out of a non-binding framework [18]. However, Rourke and colleagues stress that SMTA 2 does not create direct or binding obligations between supplier member states and third-party users of biological PIP material, raising legitimate concerns about the effectiveness of ABS [15]. Setting the WHO as a central broker, the PIP framework offers an existing infrastructure with ABS principles, which might be extended to all pandemic paGR (not only influenza ones) [19]. At this stage, the PIP Framework’s contribution to the Pandemic Treaty negotiations was mostly limited to providing a set of definitions and agreed language that may facilitate framing the process. Still, the May 2024 draft versions appear to have more obvious inspirations, particularly regarding the PABS design. Maybe surprisingly, it appears that whenever the Pandemic Treaty comes into force, it may not replace or fuse with the PIP Framework but would rather run in parallel, adding to the complexity of the paGR governance (A/INB/9/3 Revision 1, Article 12 (2c)) [9].

In addition, the International Health Regulations (IHR) are complementary important instruments to global health governance and have been recently revised. [20,21]. Entered into force in 2005, IHR are a set of legally binding rules ‘to prevent, protect against, control and provide a public health response to the international spread of disease’ (IHR, Article 2) [22]. The IHR recognises that some public health situations should be considered as public health emergency of international concern and provides a ‘decision instrument’ to assess which event may require a notification to the WHO (IHR, Annex 2). An amended version of IHR has been agreed during the 77th session of the WHA in May 2024. It introduces, for example, a definition of ‘pandemic emergency’ (Article 12, A77/A/CONF/14). While it also shows some level of commitment to solidarity and equity (Article 3.1, A77/A/CONF/14), it remains very unclear how the two instruments, IHR and the Pandemic Treaty, might interact. The May 2024 INB draft calls upon ‘harmonisation, coherence and coordination’ without clear terms (A/INB/9/3 Revision 1, Article 20 (3b)). These two instruments may overlap in their scopes, but their approaches, principles and means of implementation are quite different. Indeed, the IHR fosters more executive powers to the WHO and its Director-General to manage emergencies [21]. This may include, for example, legal means to force access to materials and data without any incentive to share the resulting benefits [3,23]. IHR do not explicitly include the concept of ABS and their latest revision still raises questions about the efficacy of such measures to build trust across the pandemic divide [3,23].

It is, therefore, essential to bear in mind that the Pandemic Treaty negotiation is not performed in an institutional vacuum but navigates at least between these major instruments, the CBD, the PIP, and the IHR. How the new Pandemic Treaty might interact with the current framework remains to be determined. Some elements, such as how the PIP deals with genomic data, could offer interesting avenues for drafting the new treaty. Having set the legal context in which the Pandemic Treaty negotiations are occurring, we highlight three major points to ensure the products of these negotiations will be on target: the scope, the modality of a PABS system and the consequences of digitisation of paGR. We then warn against the danger of bringing a new instrument into a busy and fragmented regime complex [21]. Finally, we recommend that a major effort to keep science onboard is necessary, and creating a dedicated scientific committee could be very helpful in that regard.

## A PRECISE SCOPE IS ESSENTIAL TO THE PANDEMIC TREATY’S EFFICIENCY AND RELEVANCE

The May 2024 INB’s version of Article 2 of the Pandemic Treaty draft is titled ‘Objective and Scope’ but remains pretty much an empty shell with no scope defined. It states that the Pandemic Treaty shall be ‘guided by equity and the principles further set forth herein, is to prevent, prepare for and respond to pandemics’ (A/INB/9/3 Revision 1, Article 2). Indeed, the Pandemic Treaty does not explicitly address the diversity, quality or number of pathogens causing pandemics that would fall under its regulation. In its first speech to the INB in March 2022, the WHO Director-General referred to ‘all pathogens’ (A/INB/1/4 Revision 1), but the draft versions published by the INB remain unprecise. For example, since November 2023, INB draft Articles 4–5 mention antimicrobial resistance in the Pandemic prevention and surveillance and One Health approach. This might imply that the Pandemic Treaty scope also includes antimicrobial resistance. To our understanding, while antimicrobial resistance is dramatic for global public health and sometimes also considered a pandemic (*Yersinia pestis* is indeed a bacteria), the extent to which antimicrobial resistance would be considered in the scope of the treaty draft remains unclear [24]. The concept of ‘pathogen with pandemic potential’ in the May 2024 INB draft (A/INB/9/3 Revision 1, Article 1f) is new and fluctuating, with many brackets included. It may stand as ‘any pathogen that has been identified to infect a human, and that is novel (not yet characterised) or known (including a variant of a known pathogen), potentially highly transmissible and/or highly virulent with the potential to cause a public health emergency of international concern.’ Defining precisely this term will have consequences on the scope of the Treaty and the obligations of its signatories. A narrow scope, like typically differentiating non-pandemic and pandemic strains from a taxon of potential concern or limiting it to emergency declarations, can seriously impair pandemic preparedness. For example, efficient influenza pandemic prevention requires a survey of non-pandemic influenza strains that are likely to recombine genomes and become potentially pandemic [23].

Precisely defining the scope of the Pandemic Treaty is essential to ensure its effectiveness and legal certainty. It will also be important to determine whether the resulting PABS system constitutes a specialised international agreement (NP, Article 4) and how the claimed consistency with other existing legally binding instruments will be articulated. These considerations should address technical difficulties in predicting the pandemic potential of pathogens, as evidenced by the Severe Acute Respiratory Syndrome-Coronavirus-2.

An additional aspect that has partially been eluded in the various versions of the drafts is the growing concerns over data governance originating from paGR, mostly from a complex ecosystem of technologies referred to as omics technologies that are produced and stored under various digital forms. Currently, two acronyms are used to refer to concepts that are slightly overlapping. Genomic Sequence Data (GSD), which is defined in the PIP Framework as the ‘order of nucleotides found in a molecule of DNA or RNA’. GSD is used exclusively in the context of the PIP framework (it appeared in earlier versions of the INB drafts and was eventually replaced by ‘digital sequences’). A more commonly used term, Digital Sequence Information (DSI), is mostly used under the remits of the CBD and other ABS instruments [25]. While not being defined to date, DSI has been used as a placeholder since 2016 in most ABS negotiating fora (see below for details) [26]. Many possible definitions have been proposed for DSI: sensu stricto, it would only include DNA and RNA (i.e. GSD) but sensu largo could include proteins, metabolites or even other phenotypic, structural and meta-data [26,27].

Using a particular terminology may have a real impact on the scope of the discussion and mechanically on the new instrument’s provisions. A good example of the impact of a potentially restrictive definition of DSI is prions. Prions are responsible for some zoonoses like the Creutzfeldt-Jacob disease. Being only constituted of proteins, i.e. no DNA or RNA, it would fall out of the scope of a GSD or DSI sensu stricto definitions, although being a very serious threat to global health that surely would be relevant for the Pandemic Treaty. Therefore, access to genetic sequence is necessary but not always sufficient. For certain pathogens with pandemic potential, pathogenic fungi, for example, access to strains and not only sequence data are needed to find effective treatments. Defining a relevant and precise scope for the Pandemic Treaty to be enforced should account for the structures of biobanks and the complexities associated with a narrow approach. Collections of pathogens, tangible biobanks and digital databases often operate with an extensive rationale, generally centred around multiple types of organisms. For instance, the European Virus Archive coordinates 38 laboratories and over 1900 virus strains, including chikungunya, influenza A, Midde East respiratory syndrome coronavirus, Ebola, Zika, and Severe Acute Respiratory Syndrome Coronavirus 2 [28]. Similarly, the European Molecular Biology Laboratories recently released an open access ‘pathogens portal’ that gathers data on over 200 000 pathogen species [29].

The scope question is central and must be addressed to avoid misunderstanding and false hopes. Because of the complexity of defining an accurate scope and its possible consequences regarding infrastructure and funding, a detailed discussion on the exact boundaries of the Pandemic Treaty should be held. Considering biobank structures and the challenges associated with predicting the pandemic potential of different agents, it could be argued that the scope of the Pandemic Treaty should be maintained as broad as possible. Possible options could be examined further: pathogenic viruses/pathogens, zoonotic pathogens, any viruses, any pathogens involved in a public health emergency of international concern, any pathogens including bacteria and fungi, or only some pathogens depending on their biosafety grade or global health relevance. While some options do not exclusively focus on obvious pandemic-related strains, it may remain interesting to consider building a global pathogen commons. By adopting a similar approach to the International Treaty on Plant Genetic Resources for Food and Agriculture [30,31], the Pandemic Treaty could integrate a list of specific pathogens or categories of pathogens that the treaty would cover, and the list could be reviewed. However, in the context of pathogens, care should be taken not to limit this common pool of resources – an open-ended list of properties/descriptors common to any pathogenic material, such as viruses, bacteria or even prions, could be a good alternative. In any case, setting the scope will require careful weighing of the interests, closely monitored by the relevant experts to deliver an efficient instrument.

## BEYOND ACCESS AND BENEFIT-SHARING FOR PATHOGENS

Access and benefit-sharing is part of a complex and fragmented regime for biodiversity conservation and use, whose limits must be addressed before even considering translating it to other sectors (WHO and the biodiversity regime complex; personal communication with Switzer S, 2023). Originally inspired by the CBD, the principles of equity and fairness brought forward by the PIP framework seem central and could form the backbone of the Pandemic Treaty. Inspiration from other sources (International Treaty on Plant Genetic Resources for Food and Agriculture, United Nations Convention on the Law of the Sea on the conservation and sustainable use of marine biological diversity of areas beyond national jurisdiction, etc.) is important at a time when negotiations suffer from a strong path-dependency to ABS in its Nagoya form (WHO and the biodiversity regime complex; personal communication with Switzer S, 2023) [2,31,32]. For some, the NP’s bilateral approach of ABS is outdated and ineffective in dealing with modern scientific and public health issues, particularly regarding the governance of genomic data like GSD [10,33,34]. Carefully observing the different evolutions of the Pandemic Treaty drafts, some aspects are not clear, notably a semantic shift between ‘access’ and ‘benefit-sharing’ and what may be covered by the term ‘access’ to genetic resources. In this emerging interpretation, giving access to genetic resources (via, for example, the publication of genomic data on openly accessible databases) is already considered non-monetary benefit-sharing. This confusion between access and benefit-sharing is relatively surprising, given the long history and the strong legal bounds that define ABS. Due to the ease of access associated with digitising genetic resources, some players present open access to DSI as an important form of non-monetary benefit-sharing [35]. This may be problematic on two counts. First, the issue of access to digitised genetic resources is generally sidestepped, presenting it only as ‘benefit-sharing,’ and therefore removing the ‘access’ political knot from the negotiation game. In other words, sharing data are not to be confused with benefit-sharing. On a very similar line, while facilitated access may be one of the objectives of the Pandemic Treaty, it should not replace the possibilities to foster equity via the sharing of benefits from using paGR and their associated data. Second, this semantic shift overlooks the digital divide and the massive differences in capabilities between parties, which do not reflect the possibility that some actors may not benefit from DSI likewise [2,36]. Therefore, promoting open access may not legitimately be considered fair by all actors. Not all actors can use open-access results and scientific publications, create new drugs, and manufacture and commercialise them [10]. Interestingly, the exact modalities of the PABS have been gradually fleshed out during the negotiations. For the first half of 2024, the only provisions remaining in Article 12 are those addressing the establishment of a PABS system which modalities would only be defined at a later stage, i.e. after the Treaty’s signature and left to the conference of the parties to decide (A/INB/9/3 Revision 1, Article 12). In other words, parties would adopt an empty shell with some guiding principles which flesh should be negotiated later.

Given the fragmented nature of ABS, drafting the Pandemic Treaty could benefit from the inspiration of other solutions and the consideration of difficulties encountered [37,38]. Indeed, the effectiveness of a legally binding instrument depends not only on its formal nature but also on a set of control and compliance mechanisms and on the good faith of the member states that wish to respect their obligations [39]. Providing ways for an instrument like the Pandemic Treaty to ‘learn’ and to allow a certain degree of flexibility upon reviewing its mechanism regularly could be a very promising way to effectively operationalise its provisions.

An additional recommendation would be to try not to operate a semantic shift over the access to paGR. Accessing a genetic resource or GSD/DSI/digital sequence differs from sharing the benefits deriving from its use. Furthermore, while some actors present open access to genetic resources data (DSI, GSD or others) as a form of benefit-sharing, we stress that this is not a unanimously shared position. Considering the fragmentation of the ABS regime and trying to go beyond path dependence, a way beyond compensation for access to paGR through inclusion and a multilateral approach would be necessary [40-42]. Therefore, this multilateral approach, necessary to create trust, will need to go beyond a compensatory ABS, essentially based on elusive promises of capacity-building or arbitrarily determined shares of health products production.

## TRIMMING FORCES WITHIN THE ABS FRAMEWORK

Independently of the exact scope negotiated under the Pandemic Treaty, the paGR that will be regulated through this new instrument will mostly originate from the gene pool of the CBD, and its NP, hence calling for consistency and supportiveness between ABS treaties (A/INB/9/3 Revision 1, Article 12, New Para). This significant subtraction – terrestrial biodiversity minus pandemic pathogens, or more precisely, this additional layer of regulatory complexity on paGR, must be put in perspective. Increasing trimming forces are applied to the global ABS regime complex. First, in its May 2024 version, the Pandemic Treaty creates a new treaty-specific subset of genetic resources. This is not an entirely new process (this possibility is even legally acknowledged in Article 4 of the NP) but generally raises the issue of weakening the visibility and efficiency of the ABS framework [43]. Why is a new instrument necessary if it is to share the same underlying principles as the pre-existing one? Second, digitising genetic resources, be they pathogenic, pandemic, or other, also exerts tensions over the ABS framework. The many issues underlying digitisation of paGR, like the impossibility of holding a bilateral approach to benefit-sharing, have been partially anticipated in the PIP framework and might be a good source of inspiration. The further the regime complex develops, the more unlikely a unique, inter-sectorial and multilateral solution would emerge [44]. Digitisation has been one of the major bottlenecks of ABS negotiation in the last decade, and any attempt to make a deal should carefully consider the pleiotropic effects on the entire ABS regime complex [10,31,45,46].

Ignoring other ABS silos and powerplay is probably counter-productive in the long term for the credibility and efficiency of each of these treaties. While all ABS instruments dealing with genetic resources share common principles, the mutual supportiveness across instruments remains largely incantatory. Care should be taken in the Pandemic Treaty negotiations to mitigate these tensions.

## BRINGING ‘MODEST’ SCIENTIFIC ADVICE TO THE PROCESS

**Figure 1:**
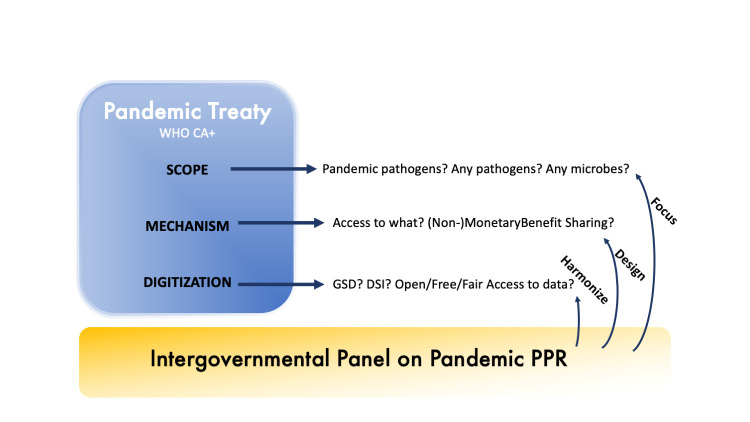
The Intergovernmental Panel on Pandemic Prevention, Preparedness and Response (PPR), embedded in the Pandemic Treaty provisions, should have three main functions: focusing the scope, designing adapted modalities to ABS and harmonising DSI/GSD policy across all mutually supportive ABS instruments. ABS – access and benefit-sharing, DSI – digital sequence information, GSD – genomic sequence data.

Negotiating a new legally binding instrument like the Pandemic Treaty is the nexus between many uncoordinated forces and interests, being social, economic, biological, epidemiological or geopolitical, yielding an unpredictable result. Considering the highly technical nature of the potential solutions to be fostered through the Pandemic Treaty to tackle the next pandemic, we advocate for a major involvement of the scientific communities in the negotiating process, drafting of the new instrument and its later development in the various subsidiary bodies possibly to emerge. Article 21.7 of the May 2024 draft allows for the treaty to establish subsidiary bodies ‘as it deems necessary’ (A/INB/9/3 Revision 1). We believe a dedicated ad hoc subsidiary body is necessary to help efficiently articulate how new scientific data and knowledge could impact the actual pandemic response and particularly the treaty inception. Drawing on the successful examples of the International Panel on Climate Change (IPCC) and the International Science-Policy Platform on Biodiversity and Ecosystem Services (), we reiterate the call for creating a group of experts at the interface between science and policy to inform and guide the complex process of pandemic preparedness and monitoring (**Figure 1**) [47,48]. An Intergovernmental Panel on Pandemic Prevention will be based on gained experience from the IPCC and IPBES and synthesise knowledge and provide recommendations [49,50]. It could help clarify the treaty’s scope based on scientific evidence and determine how to deal with omics data, or how certain benefit-sharing options would impact a pandemic [51-53]. A comprehensive and substantive involvement of civil society, indigenous population and local communities, holders of traditional knowledge, and any other relevant actor in the work would be central to such a panel. It would also need to consider encompassing a broad definition of what is defined as knowledge and by whom, i.e. have a holistic, inclusive and diverse approach to pandemic prevention, preparedness and response [54].

Philosophy and sociology of science literature denounce the instrumental use of apolitical knowledge discourse to mask political choices and positions [51,55]. Consequently, the panel’s institutional architecture and governance must be coupled with a critical perspective on the normative claims of scientific discourse [56,57]. In a very similar way to current practices in the IPBES, the formulation of policy response to pandemics must be adapted to vulnerable groups [47]. In line with a modest vision of science, this platform will attempt to provide relevant input for politicians without constraining or imposing a decision that goes beyond the political process but rather guides it [55]. This inclusive and modest perspective on science is essential in a context where, on the one hand, the Pandemic Treaty is the subject of ‘fake news’ and misinformation, and on the other hand, the WHO and its Director-General use ‘scientific’ and ‘objective’ narrative to justify the treaty; which remains a political instrument [58].

Finally, the Pandemic Treaty should be considered an opportunity to rationalise the institutional landscape and take advantage of existing infrastructures. Any scientific advisory committee should build upon various expert groups that are navigating global health governance. Typically, the High-Level Group on the One Health Initiative established by the Food and Agriculture Organization, the WHO, the United Nations Environmental Programme, and the World Health Organization for Animal Health already provides policy recommendations. Other groups like the panel for the global public health convention or the international network for evaluation of One Health may also be considered to contribute to shaping a coherent advisory body.

## CONCLUSIONS AND RECOMMENDATIONS

Taking advantage of two decades of observing other ABS-related sub-sectors dealing with surprisingly comparable issues, we recommend for the Pandemic Treaty to define ex ante a clear scope; carefully consider the peculiarities of the PABS; and mitigate the institutional fragmentations [59]. While the CBD is often considered an umbrella treaty for genetic resources, we argue that any further development considering actual or yet-to-be pandemic pathogens would require some degree of mutual support and coordination across sectors (WHO and the biodiversity regime complex; personal communication with Switzer S, 2023) [40]. The Pandemic Treaty could then be an opportunity to seize this shift towards equity in Global Health: aiming to share pathogen samples and data in alignment with the principles of fair and equitable access and benefit-sharing. Ultimately, a common goal would be striving to improve the practices during the COVID-19 pandemic, where sharing was performed vastly without legal oversight [4]. Yet, many issues are related to operationalising equity and dealing with public health crises, such as the production and distribution of countermeasures, technology transfer and access to data and intellectual property matters. Notwithstanding the degree of complexity and technicality associated with modern genomics, it remains clear that a significant participation of science in drafting an efficient instrument is necessary. Improving the way science can inform the Pandemic Treaty provision as a prerequisite to its efficiency, while probably insufficient to overcome a potential lack of political will. Compared to other ABS instruments, the Pandemic Treaty concentrates many anthropocentric and ethical concerns that push the stakes in different dimensions. Our recommendations aim to help answer this once-in-a-generation challenge and bring both equity and science to the forefront.
